# Modifications of the Prothrombin Active Site S4 Subpocket Confer Resistance to Dabigatran

**DOI:** 10.1055/a-2537-6037

**Published:** 2025-03-24

**Authors:** Viola J.F. Strijbis, Ka Lei Cheung, Dejvid Veizaj, Tessa Rutten, Boris de Bruin, Pieter H. Reitsma, Daniël Verhoef, Mettine H.A. Bos

**Affiliations:** 1 Division of Thrombosis and Hemostasis, Department of Internal Medicine, Einthoven Laboratory for Vascular and Regenerative Medicine, Leiden University Medical Center, Leiden, The Netherlands; 2 VarmX B.V., Leiden, The Netherlands

**Keywords:** anticoagulants, direct thrombin inhibitors, anticoagulant reversal agents, serine proteases, recombinant fusion proteins

## Abstract

**Background:**

Direct anticoagulants inhibit coagulation serine proteases by reversibly engaging their active site with high affinity. By modifying the S4 active site subpocket of factor (F)Xa, we introduced inhibitor resistance while preserving catalytic activity. Given the homology between FXa and thrombin in active site architecture and direct anticoagulant binding, we have targeted the S4 subsite to introduce inhibitor resistance in (pro)thrombin.

**Methods:**

Recombinant prothrombin variants were generated in which I174 was substituted or sequence R92-N98 was exchanged with that of human kallikrein-3.

**Results:**

Specific prothrombin clotting activity of the variants was 6-fold (intrinsic clotting) to 10-fold (extrinsic clotting) reduced relative to wild-type prothrombin. Further analyses revealed that modification of the S4 subsite hampers fibrinogen and thrombomodulin-mediated protein C conversion by thrombin. Consistent with this, the thrombin variants displayed a reduced catalytic efficiency toward the peptidyl substrate used in thrombin generation assessments. The variants displayed a 2-fold reduced sensitivity for dabigatran relative to wild-type prothrombin, while argatroban inhibition was unaffected. Analyses using a purified component system revealed an up to 24-fold and 4-fold reduced IC _50_ for inhibition of thrombin by dabigatran and argatroban, respectively. Molecular dynamics (MD) simulations of both dabigatran-bound and unbound (apo) modified thrombin variants indicated these to comprise a larger inhibitor binding pocket relative to wild-type thrombin and display reduced inhibitor binding. As a net effect, (pro)thrombin variants with S4 subsite modifications supported detectable fibrin formation at therapeutic dabigatran concentrations.

**Conclusion:**

Our findings provide proof-of-concept for the engineering of thrombin variants that are resistant to direct thrombin inhibitors by modulating the S4 subsite.

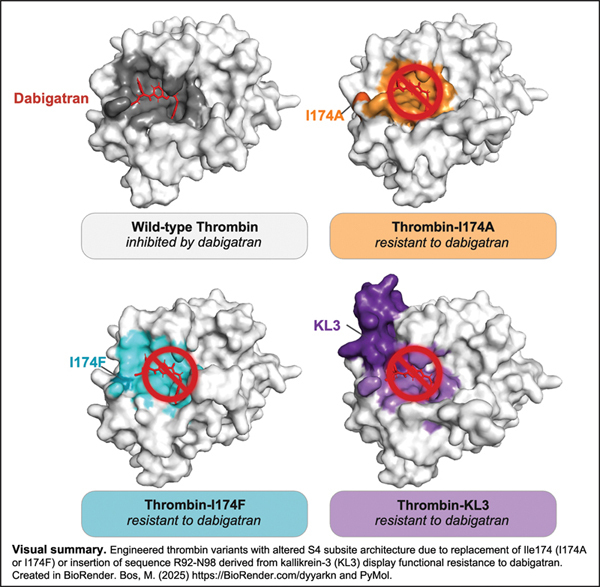

## Introduction

Chymotrypsin-like serine proteases are central to the coagulation system as they catalyze reactions that effectuate the generation, preservation, and final dissolution of a fibrin clot. The protease domain of serine proteases is hallmarked by the catalytic triad residues His57 _c_ , Asp102 _c_ , and Ser195 _c_ ( _c_ for chymotrypsinogen numbering 1 ) all positioned in the active site cleft. The catalytic triad and associated oxyanion hole residues govern substrate cleavage, while the active site subpockets (S1–4) control substrate recognition and binding. 2 The remarkable structural homology of serine proteases allows for analogous strategies in drug design. This is best underscored by the mechanism of action of several orally active, synthetic serine protease inhibitors, the so-called direct oral anticoagulants (DOACs) that selectively target factor (F)Xa (e.g., rivaroxaban, apixaban, and edoxaban) or thrombin (e.g., dabigatran). These DOACs find utility in the prophylactic management of stroke in atrial fibrillation and in the prevention and treatment of venous thrombosis. 3 4 5 6 7 8 9 10 11 12 13 Their mechanism involves reversible, high-affinity binding to the FXa or thrombin active site, thereby impeding the coagulation process. 14 15 16 17 One of the major ongoing concerns is the management of DOAC-associated bleeding complications. Hemorrhages linked to DOACs can be managed using non-specific or specific reversal agents, as comprehensively reviewed earlier by us and others. 18 19 Currently, andexanet alfa and idarucizumab are the only specific agents approved for the reversal of direct FXa inhibitors and the direct thrombin inhibitor dabigatran, respectively. 20 21

To expand the repertoire of DOAC-specific reversal strategies, we have previously successfully modified the S4 subsite of FXa by either substituting S4 subsite residue Phe174 _c_ or inserting specific sequences in the 99 _c_ -loop directly adjacent to the S4 subsite. 22 23 These modifications effectively hindered FXa active site engagement by rivaroxaban, edoxaban, and apixaban up to nearly 800-fold while preserving catalytic activity. 22 23 Given the high similarities of FXa and thrombin in both active site architecture and subsites targeted by the DOACs, 16 we have introduced modifications at or in close proximity to the S4 subsite in thrombin. These modifications include either singular amino acid substitutions at residue Ile174 _c_ or 99 _c_ -loop insertions of sequences varying in length derived from other chymotrypsin-like serine proteases. The latter include human kallikrein-related peptidases kallikrein 3 (KL3) and kallikrein 10 (KL10) and an isoform of FXa (ISO10) from the common brown snake *Pseudonaja textilis* ; all comprise elongated 99 _c_ -loop sequences that modulate the ligand specificity of the active site. 22 24 25 26 27 28

The objective of this proof-of-concept study is to determine if the same approach previously employed for the development of DOAC-resistant FXa variants can be applied to the serine protease thrombin. Here we aim to establish whether modulation of the S4 subsite leads to thrombin variants that exhibit resistance to direct thrombin inhibitors.

## Methods

### Human Plasma

Normal pooled human plasma (NPP) comprising platelet-poor plasma from 20 or more male and female donors (18–66 years) was obtained from Precision Biologic. Prothrombin-immunodepleted human plasma was from Stago.

### Reagents

All tissue culture reagents were from Thermo Fisher Scientific, and the Q5 ^®^ Site-Directed Mutagenesis Kit and restriction enzymes from New England Biolabs. Phospholipid TGT containing phosphatidylserine, phosphatidylcholine, and sphingomyelin was from Rossix. Dabigatran was from TargetMol and dissolved in 1 M HCl to 10 mg/mL. Argatroban (Arganova monohydraat) was from Goodlife Pharma and obtained as a 100 mg/mL solution containing 400 mg/mL EtOH and 300 mg/mL sorbitol. All functional assays were performed in Hepes-buffered saline (HBS: 20 mM Hepes, 0.15 M NaCl, pH 7.5) supplemented with 0.1% (w/v) PEG8000 (dilution buffer) and 5 mM CaCl _2_ (assay buffer), unless otherwise stated.

### Proteins

Human plasma-derived prothrombin, protein C, α-thrombin, activated factor X (FXa), and fibrinogen were from Prolytix; recombinant human thrombomodulin was from Protein Specialists. Recombinant constitutively active B-domainless human factor V (FV-810) 29 was expressed in BHK cells in large scale; conditioned media was collected and FV-810 was purified as described. 22 Molecular weights (Da) and extinction coefficients ( *E*_0.1%_ , 280 nm) of the proteins used were as follows: prothrombin, 72,000 and 1.38; α-thrombin, 37,500 and 1.94; fibrinogen, 340,000 and 1.51; FXa, 46,000 and 1.16; FV-810, 154,000 and 2.16. For the prothrombin variants all values for the non-modified protein were used.

### Construction and Expression of Recombinant Prothrombin

**Fig. 1:**
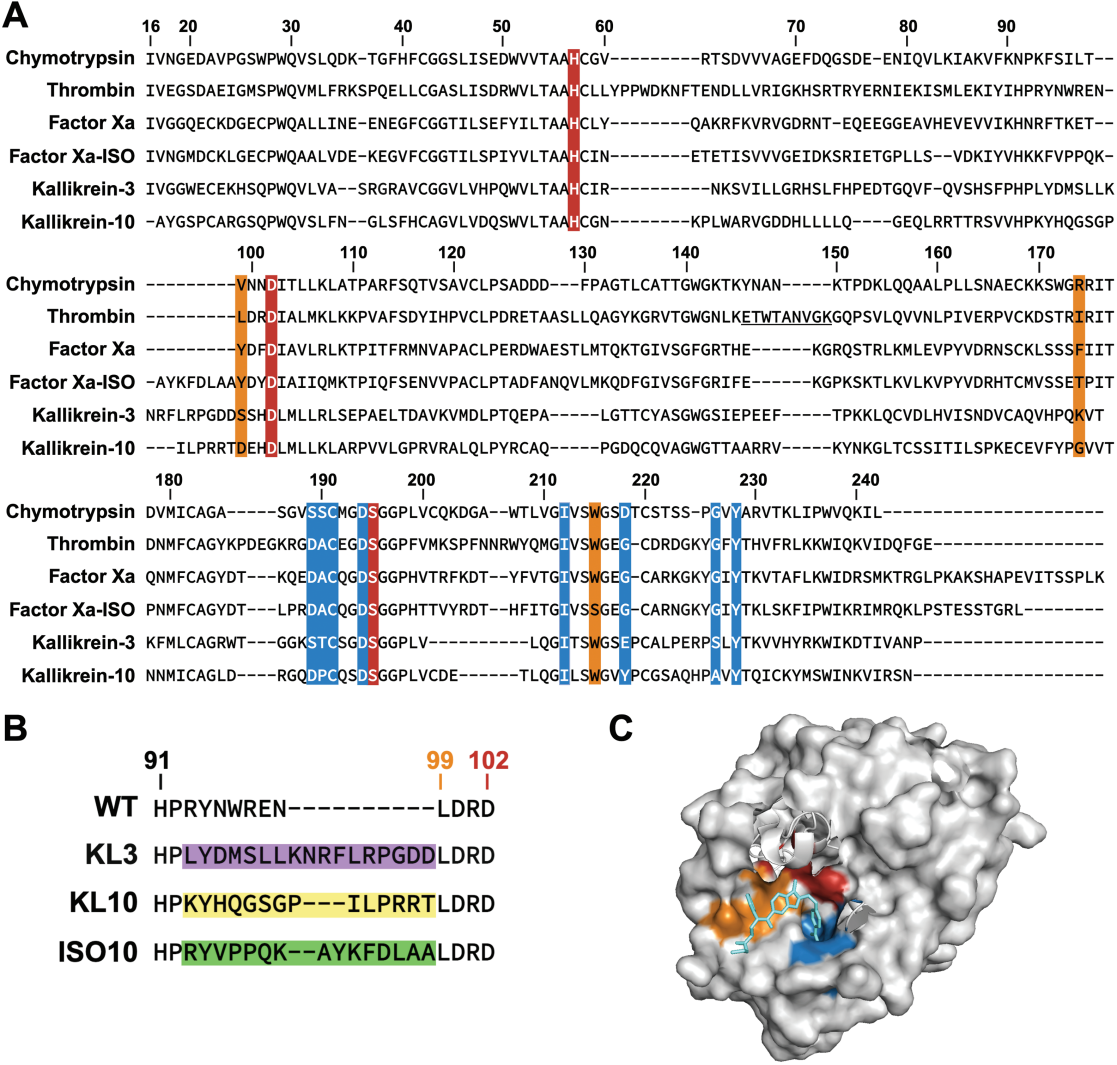
Conservation and architecture of the thrombin active site region. ( **A** ) Sequence alignment (Clustal Omega) of the serine protease domains of chymotrypsin B (Chymotrypsin, UniProt P17538), thrombin (UniProt P00734), FXa (Factor Xa, UniProt P00742), FXa isoform 2 from *Pseudonaja textilis* (Factor Xa-ISO, UniProt Q1L658), kallikrein-3 (also known as prostate-specific antigen, UniProt P07288), and kallikrein-10 (UniProt O43240). The 148 _c_ -loop, also known as the γ-loop or autolysis loop, in thrombin is underlined. ( **B** ) Sequence alignment of region His _c_ 91-Asp _c_ 102 in human prothrombin (WT), prothrombin-KL3 (KL3), prothrombin-KL10 (KL10), and prothrombin-ISO10 (ISO10). The sequences inserted between His _c_ 91 and Tyr _c_ 99 originating from kallikrein-3 (purple), kallikrein-10 (yellow), or FXa isoform 2 from *Pseudonaja textilis* (green) are shown. ( **C** ) Surface representation of the thrombin serine protease domain in complex with dabigatran (in aqua) (PDB ID 1KTS) created with PyMol. In panels **A** and **C** active site regions are indicated as follows: catalytic residues His _c_ 57, Asp _c_ 102, and Ser _c_ 195 in red; S1 subsite, also known as specificity pocket, residues 189 _c_ -191 _c_ , 194 _c_ , 212 _c_ , 218 _c_ , 226 _c_ , and 228 _c_ in blue; S4 subsite residues 99 _c_ , 174 _c_ , and 215 _c_ in orange. Created in BioRender. Bos, M. (2025) https://BioRender.com/z28s2ix and PyMol.

The DNA construct encoding for human wild-type prothrombin (prothrombin-WT) was synthesized by GenScript and subcloned into the pcDNA3.1+ vector using NheI and *Eco* RI and T4-DNA ligase. DNA constructs encoding human prothrombin variants comprising the KL3, KL10, or ISO10 modifications ( Fig. 1A, B ) were synthesized by GenScript and subcloned into the pcDNA3.1_prothrombin-WT vector using AarI and T4-DNA ligase. The I174A or I174F substitutions were prepared from pcDNA3.1_prothrombin-WT by site-directed mutagenesis. All DNA constructs were sequenced for consistency. Human Embryonic Kidney 293 (HEK293, CRL-1573; ATCC) cell lines stably expressing recombinant human prothrombin variants were obtained following transfection of pcDNA3.1_prothrombin variant vectors employing Lipofectamine ^TM^ 2000 as per the manufacturer instructions and essentially as described previously for FX. 22 In brief, prothrombin expression of transfectants was assessed by conditioning individual clones for 24 hours in Dulbecco's modified Eagle's medium/F-12 without phenol red supplemented with 2 mM L-glutamine, 100 U/mL penicillin, 0.1 mg/mL streptomycin, 0.25 μg/mL amphotericin B, 100 μg/mL geneticin, 10 μg/mL insulin-transferrin-sodium selenite, and 6 μg/mL vitamin K (Konakion ^®^ , Roche) (prothrombin-specific expression media) and subsequently measuring the prothrombin-specific PT clotting activity in a modified one-step assay by mixing conditioned media with prothrombin-depleted human plasma in a 1:1 ratio. A reference curve of NPP serially diluted in assay buffer with 0.1% bovine serum albumin (BSA) mixed in a 1:1 ratio with prothrombin-depleted human plasma was used to calculate the equivalent prothrombin units per mL plasma, with 1 mL of NPP comprising 1 unit of prothrombin activity. Transfectants with the highest prothrombin expression were expanded into 6,320 cm ^2^ cell factories that were pretreated with poly-D-lysine hydrobromide (5 mg for 1 hour at ambient temperatures; Sigma–Aldrich) and conditioned for 24 hours in prothrombin-specific expression media. Conditioned media was collected for 10 consecutive days, filtered over an 0.45 μm polyethersulfone membrane, and supplemented with 1 mM benzamidine (Sigma–Aldrich) prior to storage at −20°C.

### Purification of Prothrombin Variants

Conditioned media (15 L) was thawed at 37°C, filtered over an 0.45 μm polyethersulfone membrane, applied to a size 6 A ultrafiltration hollow fiber cartridge using an Äkta flux 6 instrument (Cytiva), diafiltrated to approximately 500 mL in HBS, and stored at −20°C. Following thawing at 37°C, the concentrate was applied at ambient temperatures to a CaptureSelect Prothrombin affinity matrix (Thermo Scientific) equilibrated in 50 mM Tris, 5 mM CaCl _2_ , 0.15 M NaCl, pH 7.0. Following washing with 50 mM Tris, 5 mM CaCl _2_ , 1 M NaCl, pH 7.0, bound protein was eluted with 50 mM Tris, 5 mM EDTA, 0.15 M NaCl _2_ , pH 7.0. Fractions containing prothrombin activity were analyzed employing SDS-PAGE analysis, stored at −80°C, pooled upon thawing at 37°C, precipitated by addition of 0.516 gr/mL ammonium sulfate (Sigma-Aldrich) by stirring and overnight incubation at 4°C, collected by centrifugation (10,000 × *g* for 30 minutes at 4°C), dissolved in HBS and 50% (v/v) glycerol, and stored at −20°C. Purified prothrombin variants were visualized by SDS-PAGE analysis employing the MES buffer system followed by Quick Coomassie Stain (Protein Ark). Comparison of recombinant wild-type prothrombin protein preparations purified employing a combination of anion-exchange and hydrophobic affinity chromatography (“classic” method, 22 see Supplemental Methods S1 (available in the online version only)) versus CaptureSelect Prothrombin affinity chromatography indicated that: (i) although the overall profile of low abundant protein fragments varied, the CaptureSelect method resulted in protein preparations comprising a lower amount of additional protein fragments based on SDS-PAGE analysis ( Supplemental Fig. S1A, B [available in the online version only]), and (ii) the CaptureSelect method selects for fully γ-carboxylated prothrombin based on specific clotting activity values that are in the same range ( Supplemental Fig. S1C [available in the online version only]). The typical yield of fully γ-carboxylated recombinant prothrombin was 0.11 to 1.43 mg/L conditioned medium, depending on prothrombin variant expression.

### Specific Clotting Activity

The specific prothrombin extrinsic clotting activity was determined using a modified prothrombin-specific prothrombin time (PT) assay. Prothrombin-depleted plasma was spiked with 90 µg/mL prothrombin variant and serially diluted in assay buffer with 0.1% BSA. Samples (50 μL) were incubated for 1 minute at 37°C, upon which coagulation was initiated with the addition of 50 μL PT reagent (Neoplastine CI Plus; Stago), and the coagulation time was monitored using a Start4 coagulation instrument (Stago). A reference curve consisted of serial dilutions of NPP that was employed to convert clotting times to U/mg, assuming that 1 U/mL prothrombin in NPP equals 90 μg/mL prothrombin. The specific prothrombin intrinsic clotting activity was determined using a modified prothrombin-specific activated partial thromboplastin time APTT assay. Prothrombin-depleted plasma was spiked with 90 µg/mL prothrombin variant and serially diluted in Owren–Koller buffer (isotonic saline; Stago). Samples (50 µL) were added to 50 µL of APTT reagent (TriniCLOT; Stago), followed by a 3 minutes incubation period at 37°C. Coagulation was initiated with the addition of 25 µL 25 mM CaCl _2_ , and the coagulation time was monitored. Reference curves consisted of serial dilutions of NPP in Owren–Koller buffer, upon which the same procedure was followed as described for the prothrombin-specific PT clotting activity.

### Calibrated Automated Thrombography

Thrombin generation was adapted from protocols using low plasma volumes as previously described. 30 31 All analyses involved supplementation of prothrombin-depleted plasma with 90 or 180 µg/mL prothrombin variant assessed in the absence (same volume of dilution buffer) or presence of dabigatran or argatroban (0.01–15 µM, final concentrations). The final reaction volume was 60 µL, of which 40 µL was supplemented plasma, 10 µL PPP reagent (5 pm TF together with 4 μM phospholipids, final concentrations; Thrombinoscope), and 10 µL FluCa mix (0.45 mM fluorogenic substrate Z-Gly-Gly-Arg-AMC and 17 mM CaCl _2_ , final concentrations; Thrombinoscope). Thrombin formation was determined every 20 seconds for 120 minutes and corrected for the calibrator (Thrombinoscope) using Thrombinoscope software. The lag time, thrombin peak, endogenous thrombin potential (ETP), time to peak (TTP), and velocity index (VI) were calculated from duplicates of at least two individual measurements.

### Prothrombin Activation

Prothrombin variants were activated to thrombin by prothrombinase immediately prior to analyses. Reaction mixtures of prothrombin variant (1 μM) and preassembled prothrombinase (2 nM FXa, 2 nM FV-810, and 50 μM phospholipid TGT, final concentrations) were incubated for 30 minutes at 25°C in assay buffer, upon which the reaction was quenched in dilution buffer supplemented with 50 mM EDTA. Full conversion to thrombin was confirmed by SDS-PAGE analysis ( Supplemental Fig. S2 [available in the online version only]).

### Peptidyl Substrate Hydrolysis

The kinetics of peptidyl substrate hydrolysis were measured in assay buffer using increasing concentrations (2.5–200 μM) of fluorescent substrate Z-Gly-Gly-Arg-AMC,HCl (Z-GGR-AMC; Bachem) or chromogenic substrate H-D-Phe-Pip-Arg-pNA,2HCl (pNAPEP-0238; Cryopep) and initiated with 5 nM prothrombinase-activated prothrombin variant. Inhibition of 5 nM prothrombinase-activated prothrombin variant was assayed in the absence or presence of the direct thrombin inhibitor dabigatran or argatroban (0.0001–10 μM, final concentrations) and 50 μM pNAPEP-0238.

### Protein C Activation

Thrombin–thrombomodulin complexes were allowed to form by incubating 1 nM prothrombinase-activated prothrombin variant with 50 nM recombinant human thrombomodulin for 15 minutes at 37°C in assay buffer, followed by pre-incubation with protein C for 15 minutes at 37°C. Upon addition of peptidyl substrate H-D-Ile-Pro-Arg-pNA,2HCl (pNAPEP-1588; Cryopep), activated protein C formation was monitored over time at 405 nm and 37°C. Final concentrations of the reactants were 0.2 nM prothrombinase-activated prothrombin variant, 10 nM recombinant human thrombomodulin, 150 nM protein C, and 250 μM pNAPEP-1588.

### Fibrinogen Conversion

Fibrinogen conversion was determined employing a turbidity assay. Reaction mixtures contained 0.045 μM prothrombinase-activated prothrombin variant, 0.9 μM fibrinogen, and 0–1 μM dabigatran. Turbidity was measured for 1 hour at 25°C and 350 nm.

### Molecular Dynamics Simulations

Molecular dynamics (MD) simulations were performed using Amber2022. 32 33 For the thrombin–dabigatran complexes, the protein, dabigatran, and solvent (water) topologies were generated using tLeAP, 33 as described in Supplemental Table S1 (available in the online version only). Using the achieved final complex structure, I174A and I174F substitutions were introduced in the PDB file. The three-dimensional structures of non-inhibitor-bound thrombin were generated using AlphaFold. 34 35 Following two energy minimization steps, two equilibration MD simulations were run at constant temperature and volume (NVT), and at constant pressure and temperature (NPT) equilibration steps, respectively. Subsequently, the production MD simulations extended over 100 nanoseconds (of which the first 2 nanoseconds were discarded for additional equilibration) with coordinates written out to disk every 0.2 nanoseconds. Detailed simulations settings and Amber input files for MD can be found in Supplemental Table S2 (available in the online version only). The PDB preparation setup for each MD step is described in the Supplemental Methods. The solvent accessible surface area (SASA) of the binding pocket was calculated using PyMol 4.6.0, and the thrombin–dabigatran precursor (PDB ID 1KTS) was used as template. The average coordinate structure from each MD was aligned to the template. The inhibitor binding pocket was estimated as the atoms 6 Angstrom (Å) around the inhibitor binding site found in the template. Per-residue analysis was performed using the Molecular Mechanics Generalized Born Surface Area (MM/GBSA) model 36 ; the top interactive residues (with an individual interaction energy <0.1 kcal/mol) for wild-type thrombin were extracted from the bigger dataset. Prior to the per-residue analysis, the topology of thrombin and dabigatran or argatroban was extracted from their respective complex topology file using an ante-MMPBSA.py script in AmberTools23 with default bondi radii setting. The binding free energy of dabigatran or argatroban for thrombin variants was calculated using the MMPBSA.py 37 script in AmberTools using default settings with an ionic strength set to 0.15 mM.

### Data Analysis

All data are presented as mean ± 1 standard deviation and are the result of at least two to three experiments, unless stated otherwise. Kinetic data were analyzed by nonlinear regression using a four-parameter logistic function, and statistics were evaluated by one- or two-way ANOVA or *t* -test, where appropriate. All analyses were computed using GraphPad Prism software.

## Results

### Modifications in or Near the S4 Subsite Attenuate the Specific Prothrombin Clotting Activity

**Fig. 2:**
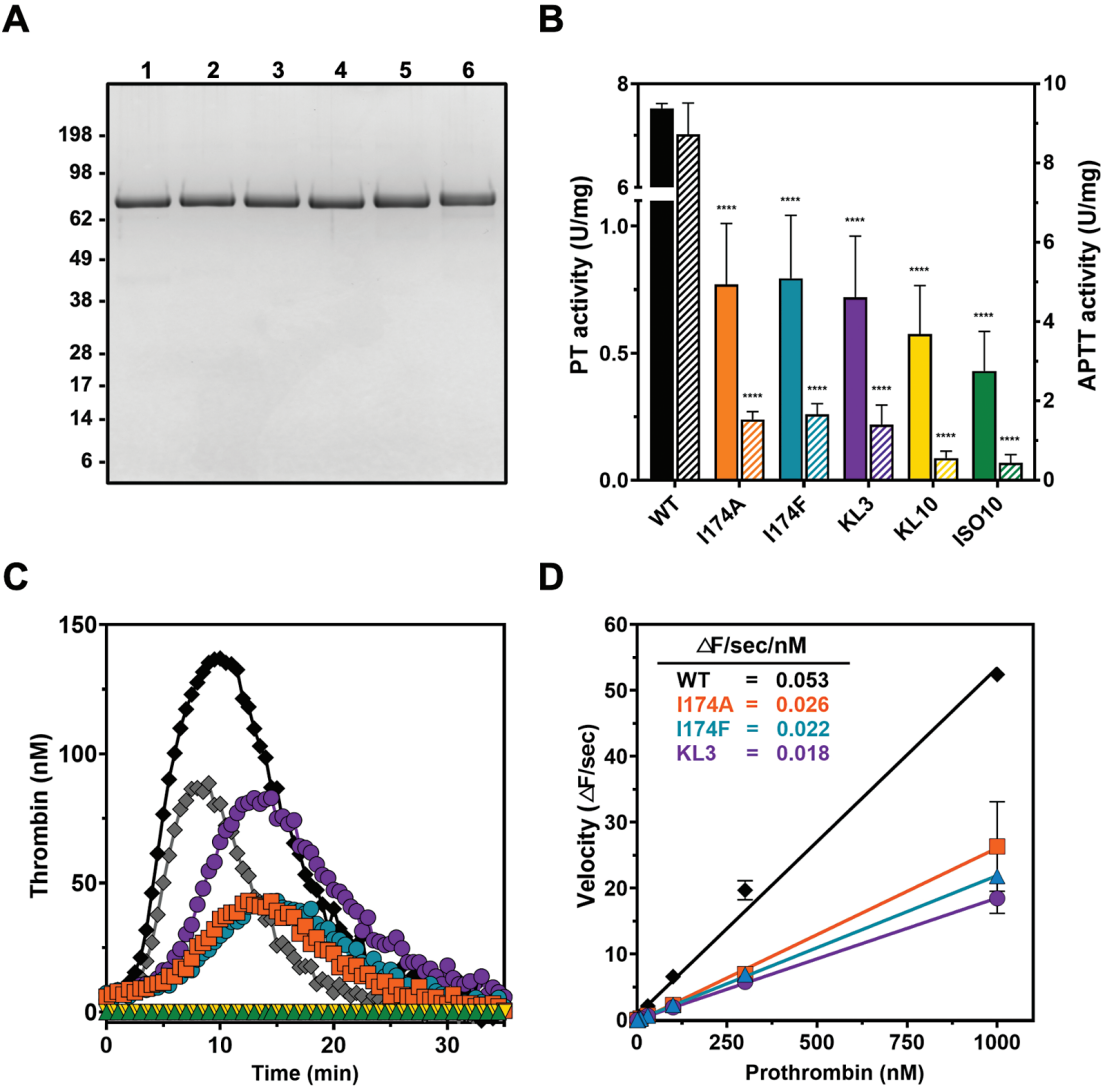
Characterization of prothrombin variants. ( **A** ) SDS-PAGE analysis of purified prothrombin variants (3 µg/lane) under reducing conditions and visualized by Coomassie staining. Lane 1, wild-type prothrombin; lane 2, prothrombin-I174A; lane 3, prothrombin-I174F; lane 4, prothrombin-KL3; lane 5, prothrombin-KL10; lane 6, prothrombin-ISO10. The apparent molecular weights (kDa) of the standard are indicated. ( **B** ) The specific clotting activity initiated by the extrinsic or intrinsic pathway employing a prothrombin-specific prothrombin time (PT)-based assay (filled bars) or prothrombin-specific activated partial thromboplastin time (APTT)-based assay (striped bars), respectively, was determined for purified wild-type prothrombin (WT) (black), prothrombin-I174A (orange), prothrombin-I174F (teal), prothrombin-KL3 (purple), prothrombin-KL10 (yellow), and prothrombin-ISO10 (green) as described in “Methods.” ( **C** ) Representative thrombin generation curves of prothrombin-depleted plasma supplemented with wild-type prothrombin at 90 or 180 µg/mL (equal to 1.3 or 2.5 μM, representing 1x or 2x the prothrombin plasma concentration, respectively) (gray or black diamonds, respectively) or 180 µg/mL of prothrombin-I174A (orange squares), prothrombin-I174F (teal triangles), prothrombin-KL3 (purple circles), prothrombin-KL10 (yellow triangles), or prothrombin-ISO10 (green triangles). Thrombin formation was triggered by the addition of PPP reagent and initiated with CaCl _2_ and fluorogenic substrate Z-GGR-AMC as detailed in “Methods.” ( **D** ) The initial velocity (change in fluorescent signal [ΔF] per second) of 0.45 mM Z-GGR-AMC conversion was assessed at increasing concentrations of prothrombin variant added to prothrombin-depleted plasma. Thrombin formation was triggered by the addition of PPP reagent, and Z-GGR-AMC conversion was monitored in the presence of 17 mM CaCl _2_ as detailed in “Methods.” The lines were drawn by fitting the data to a linear function, and the slope representing the velocity per nM prothrombin variant is shown in the inset. Mean values ± 1 standard deviation of at least two independent experiments are shown. **** *p* < 0.0001 according to one-way ANOVA analysis in comparison with wild-type prothrombin.

Following stable expression in HEK293 cells, prothrombin variants comprising modifications in or near the S4 subsite ( Fig. 1A, B ) were purified to homogeneity using the CaptureSelect Prothrombin affinity matrix that selects for fully γ-carboxylated prothrombin. SDS-PAGE analysis showed the prothrombin variants to migrate similar to wild-type prothrombin ( Fig. 2A , Supplemental Fig. S3 (online only)). Evaluation of extrinsic and intrinsic fibrin clot formation revealed that all modified prothrombin variants displayed a severe reduction in specific clotting activities, varying from a 9- to 19-fold reduction in extrinsic plasma coagulation to a 5- to 22-fold reduction in intrinsic clotting compared with wild-type prothrombin ( Fig. 2B , Supplemental Table S3 (available in the online version only)). Prothrombin-ISO10 consistently demonstrated the lowest specific clotting activity, followed by prothrombin-KL10; the specific clotting activities of the I174A, I174F, and KL3 variants were more or less similar. These findings indicate that the specific modifications introduced in or near the prothrombin active site considerably impact fibrin clot formation.

### Modifications in or Near the S4 Subsite Modify the Active Site and Impair Z-GGR-AMC Peptidyl Substrate Conversion and Protein C Conversion

Thrombin generation analyses were employed to assess the capacity of prothrombin variants to support tissue factor–initiated thrombin formation. Supplementation of prothrombin-depleted plasma with physiologically relevant concentrations of wild-type prothrombin resulted in a dose-dependent increase in thrombin generation ( Fig. 2C ). To achieve detectable levels of thrombin generation for prothrombin-I174A, prothrombin-I174F, or prothrombin-KL3, supplementation with concentrations representing 2-fold the prothrombin plasma concentration was required. While for the I174A and I174F variants thrombin generation was hallmarked by a prolonged lag time and reduced thrombin peak relative to wild-type prothrombin, prothrombin-KL3 displayed a thrombin peak in the range of wild-type prothrombin ( Fig. 2C ). In contrast, no thrombin generation was observed following supplementation with prothrombin-KL10 and prothrombin-ISO10. Considering the non-responsiveness in the thrombin generation assay and low specific clotting activities of prothrombin-KL10 and prothrombin-ISO10, these variants were not further pursued. Overall, these observations indicating that modifications in or around the S4 subsite hamper thrombin formation are in line with our findings on reduced specific clotting activity.

Subsequent assessment of the activated prothrombin-dependent conversion rate of the fluorogenic substrate Z-Gly-Gly-Arg-AMC that is used as read-out in the thrombin generation assay revealed an up to 3-fold reduced rate for conversion by activated prothrombin-I174A, prothrombin-I174F, and prothrombin-KL3 compared with wild-type ( Fig. 2D ). Assessment of the kinetic parameters of Z-GGR-AMC hydrolysis indicated a decrease in *k*_cat_ and increase in *K*_m_ for all prothrombinase-activated variants, resulting in a 3- to 5-fold reduction in the specificity constant ( Table 1 , Supplemental Fig. S4A (available in the online version only)). This suggests that modifications at or near the S4 subsite modify the active site thereby impairing Z-GGR-AMC conversion, which is at the cause of the apparent reduction in thrombin-forming potential. Employing higher concentrations of prothrombin variant overcomes this defect in part, allowing for the use of thrombin generation assays to measure thrombin inhibition by the direct thrombin inhibitors.

**Table 1:** Kinetic parameters of peptidyl substrate conversion by prothrombinase-activated prothrombin variants

	Z-GGR-AMC			pNAPEP-0238		
	*K*_m_ (µM)	*k*_cat_ (min ^−1^ )	Specificity constant ( *k*_cat/_ *K*_m_ )	*K*_m_ (µM)	*k*_cat_ (min ^−1^ )	Specificity constant ( *k*_cat/_ *K*_m_ )
**WT**	82 ± 7	14 ± 0.5	**0.17**	34 ± 13	17 ± 2	**0.49**
**I174A**	163 ± 8 a	8 ± 0.2 c	**0.05**	43 ± 14	21 ± 2	**0.49**
**I174F**	153 ± 12 a	6 ± 0.3 c	**0.04**	50 ± 15	24 ± 3 c	**0.47**
**KL3**	188 ± 13 b	5 ± 0.2 d	**0.03**	82 ± 31	35 ± 6 c	**0.43**

Abbreviations: I174A, prothrombinase-activated prothrombin-I174A; I174F, prothrombinase-activated prothrombin-I174F; k _*cat*_ , turnover number of enzyme; KL3, prothrombinase-activated prothrombin-KL3; *K*_m_ , Michaelis constant; WT, prothrombinase-activated wild-type prothrombin.

Note: ^a^ *p* < 0.05.

b *p* < 0.005.

c *p* < 0.0005.

d *p* < 0.0001 according to one-way ANOVA analysis in comparison with prothrombinase-activated wild-type prothrombin.

**Fig. 3:**
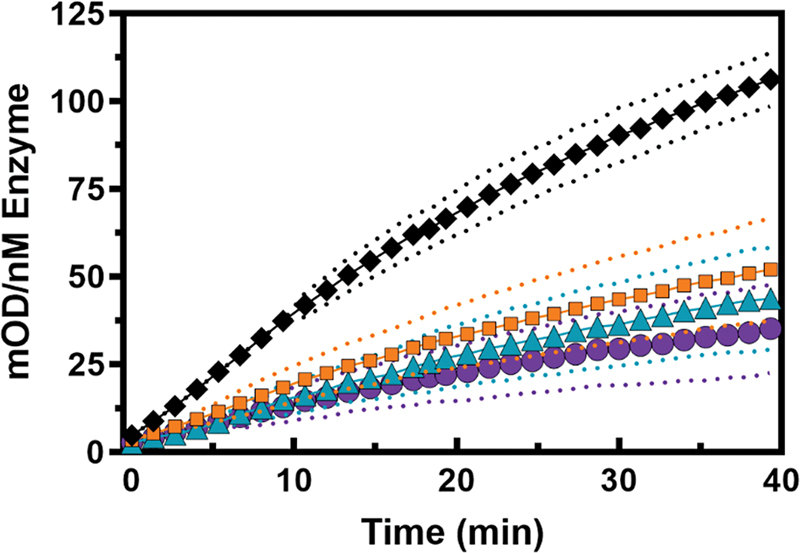
Protein C conversion by activated prothrombin variants in a purified system. Prothrombinase-activated variants (0.2 nM) were incubated with thrombomodulin (10 nM) and protein C (150 nM), upon which the rate of pNAPEP-1588 peptidyl substrate conversion by the generated activated protein C was determined. Data was corrected for thrombin-mediated pNAPEP-1588 hydrolysis, and the mean ± 1 standard deviation (dotted lines) are shown. Symbols represent prothrombin-WT (black diamonds), prothrombin-I174A (orange squares), prothrombin-I174F (teal triangles), or prothrombin-KL3 (purple circles). The data of two to three independent experiments are shown. OD, optical density at 405 nm.

To evaluate the effect of S4 subsite modifications on macromolecular substrate conversion, the thrombomodulin-mediated conversion of protein C to activated protein C was assessed. In the presence of saturating concentrations of the cofactor thrombomodulin, a reduced rate of protein C conversion by activated prothrombin-I174A, prothrombin-I174F, and prothrombin-KL3 compared with wild-type was observed ( Fig. 3 ). This indicates that modifications near the thrombin active site also affect the generation of activated protein C, thereby impairing initiation of the anticoagulant-activated protein C pathway.

### S4 Subsite Modifications Modestly Affect the Sensitivity Toward Direct Thrombin Inhibitors

**Fig. 4:**
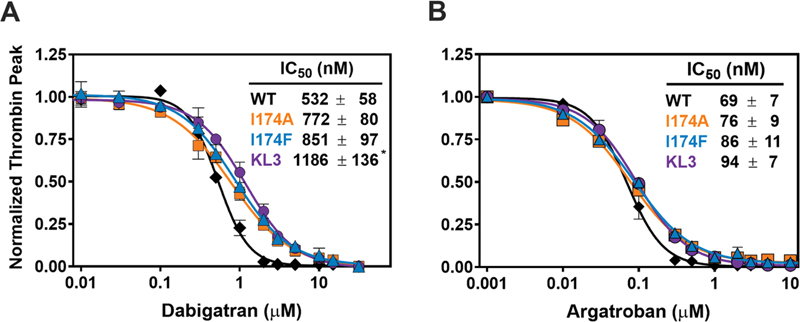
Inhibition of prothrombin variants by direct thrombin inhibitors in a plasma-based system. Thrombin generation was measured for 60 minutes at 37°C in prothrombin-depleted plasma supplemented with 180 µg/mL of wild-type prothrombin (WT, black diamonds), prothrombin-I174A (orange squares), prothrombin-I174F (teal triangles), or prothrombin-KL3 (purple circles) and increasing concentrations (0–30 µM) of dabigatran ( **A** ) or argatroban ( **B** ). Thrombin formation was triggered by the addition of PPP reagent, and Z-GGR-AMC conversion was monitored in the presence of 17 mM CaCl _2_ as detailed in “Methods.” The thrombin peak height was normalized to the peak height in the absence of inhibitor, and the lines were drawn by fitting the datasets to a four-parameter logistic function; the fitted parameters for IC _50_ ± 1 standard deviation of the induced fit are shown in the inset. Data of at least two independent experiments are shown. * *p* < 0.05 according to one-way ANOVA analysis in comparison with wild-type prothrombin.

Thrombin generation was assessed to determine inhibition by the active site–targeted direct thrombin inhibitors dabigatran and argatroban. Following supplementation of prothrombin-depleted plasma with wild-type prothrombin, the addition of dabigatran or argatroban dose-dependently decreased the thrombin peak with half-maximum inhibition at 0.5 μM dabigatran or 0.07 μM argatroban ( Fig. 4 ). Supplementation with prothrombin-I174A, prothrombin-I174F, or prothrombin-KL3 revealed a minimal trend toward a reduced inhibitor-sensitivity. The prothrombin-KL3 variant displayed the highest IC _50_ values for both direct thrombin inhibitors, with an up to 2.2-fold increase for dabigatran and 1.4-fold increase for argatroban relative to wild-type prothrombin. Taken together, these observations indicate that introduction of specific modifications at or near the S4 subsite modestly impact inhibition by the direct thrombin inhibitors in this plasma-based setup.

### Activated Prothrombin Variants Display Unperturbed H-D-Phe-Pip-Arg-pNA Peptidyl Substrate Conversion

To further assess to what extent the S4 subsite modifications affect the thrombin active site, the ability of prothrombinase-activated prothrombin variants to hydrolyze the thrombin-specific chromogenic substrate H-D-Phe-Pip-Arg-pNA (pNAPEP-0238) was determined. Whereas the I174A and I174F variants displayed kinetic parameters similar to wild-type, activated prothrombin-KL3 revealed a moderate increase (2-fold) in *k*_cat_ and *K*_m_ ( Table 1 , Supplemental Fig. S4B (available in the online version only)). Overall, all variants displayed a similar specificity constant, indicative of no net effect on the kinetics of substrate hydrolysis. These results indicate that while the active site has been modified in prothrombin-I174A, prothrombin-I174F, and prothrombin-KL3, the overall conversion of this specific peptidyl substrate remains unaffected.

**Fig. 5:**
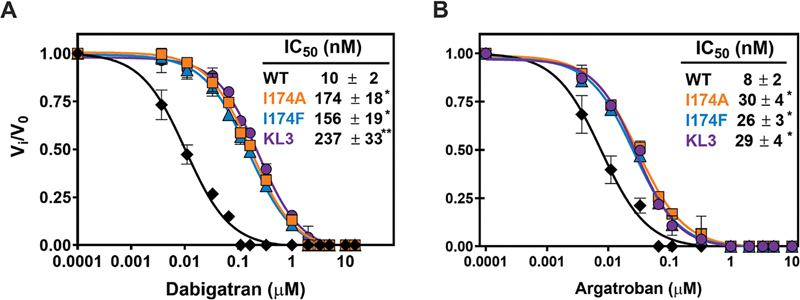
Inhibition of prothrombinase-activated prothrombin variants by direct thrombin inhibitors in a purified system. The rate of pNAPEP-0238 peptidyl substrate conversion by 5 nM of prothrombinase-activated wild-type prothrombin (WT, black diamonds), prothrombin-I174A (orange squares), prothrombin-I174F (teal triangles), or prothrombin-KL3 (purple circles) was determined in the absence (V _0_ ) or presence (V _i_ ) of increasing concentrations (0–30 µM) of dabigatran ( **A** ) or argatroban ( **B** ). The lines were drawn by fitting the datasets to a four-parameter logistic function, and the fitted parameters for IC _50_ ± 1 standard deviation of the induced fit are shown in the inset. Using the Cheng-Prusoff equation 56 the *K*_i_ values for inhibition by dabigatran were: WT, 4 nM; I174A, 80 nM; I174F, 80 nM; KL3, 150 nM; the *K*_i_ values for inhibition by argatroban were: WT, 3 nM; I174A, 13 nM; I174F, 13 nM; KL3, 17 nM. Data of at least two independent experiments are shown. * *p* < 0.05 or ** *p* < 0.005 according to one-way ANOVA analysis in comparison with prothrombinase-activated wild-type prothrombin.

Assessment of inhibition by the direct thrombin inhibitors using pNAPEP-0238 hydrolysis by prothrombinase-activated prothrombin variants as read-out revealed a 17- to 24-fold increase in IC _50_ of dabigatran inhibition ( Fig. 5A ), with activated prothrombin-KL3 being most resistant to dabigatran inhibition. Furthermore, all prothrombinase-activated prothrombin variants displayed a 4-fold increase in IC _50_ values determined for argatroban inhibition relative to activated wild-type prothrombin ( Fig. 5B ). Collectively, these data indicate that modifications in or around the S4 subsite of (pro)thrombin lead to reduced sensitivity for the direct thrombin inhibitors dabigatran and argatroban.

### MD Simulations Project Reduced Interactions of Specific Residues with the Thrombin Inhibitors Following Modulation of the S4 Subsite

**Fig. 6:**
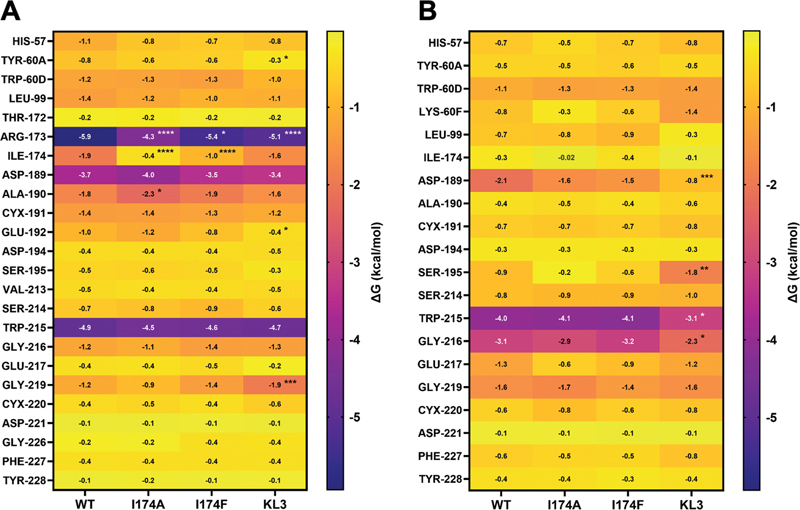
Heatmap visualization of binding free energy interactions between thrombin residues and dabigatran or argatroban. Heatmap representing MM/GBSA binding free energies (ΔG) of thrombin residues having an individual interaction energy ≤–0.1 kcal/mol with dabigatran ( **A** ) or argatroban ( **B** ), determined as detailed in “Methods.” Rows represent the wild-type thrombin (WT), thrombin-I174A (I174A), or thrombin-I174F (I174F) interactive residues indicated in chymotrypsinogen numbering. Colors indicate the per-residue MM/GBSA binding free energies (kcal/mol), scaled from <–1 kcal/mol (yellow) representing relatively weak interactions to >–5 kcal/mol (purple) indicating stronger interactions. The data represent the mean of 10 individual molecular dynamics (MD) replicates. * *p* < 0.05, ** *p* < 0.005, *** *p* < 0.0005, or **** *p* < 0.0001 according to two-way ANOVA analysis in comparison with wild-type thrombin.

MD simulations of thrombin species in complex with dabigatran or argatroban were performed to investigate the molecular mechanism of inhibition. Using the MM/GBSA approach, per-residue analyses identified specific inhibitor-interactive residues in both wild-type and thrombin variants. The MM/GBSA values of the top interactive wild-type thrombin residues defined as having an individual interaction energy ≤ –0.1 kcal/mol with dabigatran or argatroban were extracted from the thrombin variant–inhibitor complex simulations ( Fig. 6 ). Residues Arg173 _c_ , Asp189 _c_ , and Trp215 _c_ were predicted to contribute most to the interaction of wild-type thrombin with dabigatran, displaying mean MM/GBSA values ≤–3.7 kcal/mol ( Fig. 6A ). The contributions of Asp189 _c_ and Trp215 _c_ to dabigatran stabilization were retained in simulations of the modified thrombin variants, although the Arg173 _c_ contribution was projected to be significantly reduced. In addition, simulations with the I174-substituted variants predicted the 174 _c_ residue to contribute significantly less to dabigatran binding. The latter may be compensated to some extent by a moderately enhanced contribution of Ala190 _c_ to dabigatran stabilization in thrombin-I174A and thrombin-I174F. Simulations with thrombin-KL3 indicated residues Tyr60 _c_ A and Glu192 _c_ to display a significantly reduced interaction with dabigatran. The overall loss in dabigatran coordination by thrombin-KL3 was compensated by a significantly enhanced interaction of Gly219 _c_ compared with wild-type thrombin.

The interaction of wild-type thrombin with argatroban was characterized by substantial contributions of residues Asp189 _c_ , Trp215 _c_ , and Gly216 _c_ , displaying mean MM/GBSA values ≤–2.1 kcal/mol ( Fig. 6B ). While the Trp215 _c_ and Gly216 _c_ interactions were predicted to be retained in the I174-substituted variants, the Asp189 _c_ interaction was moderately reduced. Moreover, simulations of the thrombin-KL3 interactions with argatroban predicted a significantly reduced interaction of residues Asp189 _c_ , Trp215 _c_ , and Gly216 _c_ , while that of Ser195 _c_ was significantly increased.

To conclude, specificity pocket residue Asp189 _c_ and S4 subsite residue Trp215 _c_ are projected to be key interactive residues for dabigatran and argatroban stabilization. Additional dabigatran coordination is facilitated by Arg173 _c_ , while Gly216 _c_ contributes to argatroban binding. Modulation of the thrombin S4 subsite via Ile174 _c_ substitution or 99 _c_ -loop insertion impairs some of these interactions, thereby providing a rationale for the reduced inhibitor-sensitivity of these thrombin variants.

### MD Simulations Reveal That an Enlarged Binding Pocket in Thrombin Variants Leads to Reduced Inhibitor Binding

**Fig. 7:**
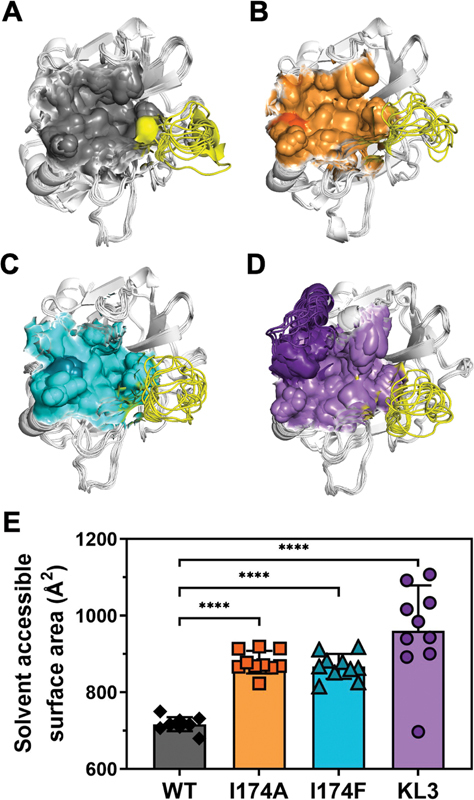
Computational analysis of the inhibitor binding pocket in thrombin variants. ( **A–D** ) Average conformations of 10 individual molecular dynamics (MD) simulations of unbound (apo) wild-type thrombin ( **A** ), thrombin-I174A ( **B** ), thrombin-I174F ( **C** ), or thrombin-KL3 ( **D** ) with the inhibitor binding pocket, determined as detailed in “Methods,” shown as a colored surface plot. The darker color represents the specific amino acid replacement (thrombin-I174A, orange; thrombin-I174F, teal) or H _c_ 91-Y _c_ 99 insertion (thrombin-KL3, purple). The 148 _c_ -loop is indicated in yellow; the sequence of the thrombin 148 _c_ -loop is indicated in Fig. 1A . ( **E** ) The solvent accessible surface area of the inhibitor binding pocket in apo wild-type thrombin (WT), thrombin-I174A (I174A), thrombin-I174F (I174F), or thrombin-KL3 (KL3) was determined from 10 individual MD apo simulations and calculated using PyMol. The data are presented as boxplots with the whiskers representing the 5th to 95th percentile of the dataset. Statistical significance: **** *p* < 0.0001 according to one-way ANOVA analysis.

MD simulations of AlphaFold-generated unbound (apo) thrombin variants were performed to determine the effect of the S4 subsite modifications on the active site structure. Analysis of the inhibitor binding pocket, comprising atoms positioned ≤6 Å from the inhibitor, indicated a significantly enlarged binding pocket following S4 subsite modifications ( Fig. 7 ). Conformational changes were noticed mostly for the 148 _c_ -loop ( Fig. 7C, D ). However, these were not driven by the modifications as wild-type apo thrombin displayed similar 148 _c_ -loop movement ( Fig. 7A ). Conversely, the size of the inhibitor binding pocket differed substantially between wild-type thrombin and the variants. Computational estimates indicated that S4 subsite modifications lead to a significant increase in solvent accessible area of the inhibitor binding pocket in apo thrombin species ( Fig. 7E ). Collectively, these computational simulations suggest that modifications at or near the S4 subsite enlarge the inhibitor binding pocket, leading to reduced inhibitor binding due to a diminished interaction of important binding residues.

### Prothrombin Variants Support Fibrinogen Conversion in the Presence of Dabigatran

**Fig. 8:**
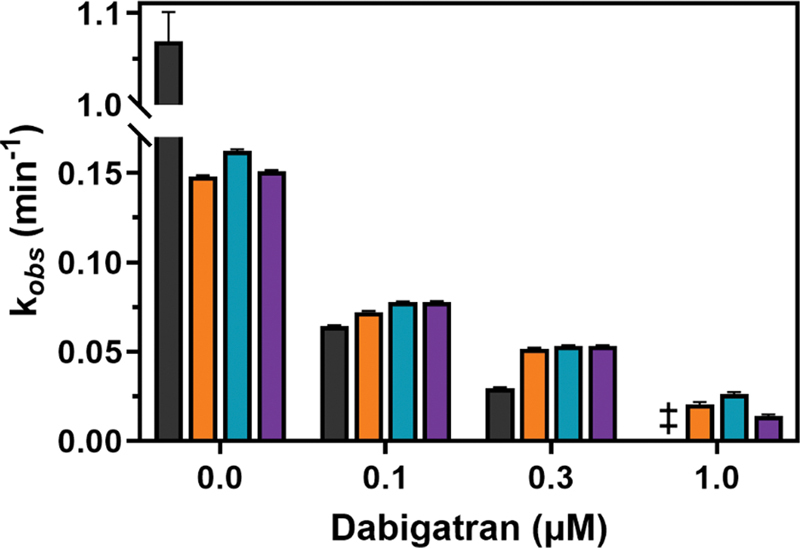
Rate of fibrinogen conversion by prothrombinase-activated prothrombin variants. Prothrombinase-activated variants (0.045 µM) prothrombin-WT (black bars), prothrombin-I174A (orange bars), prothrombin-I174F (teal bars), or prothrombin-KL3 (purple bars) were incubated with fibrinogen (0.9 µM) and dabigatran (0, 0.1, 0.3, or 1 µM, equaling 0, 65, 190, or 630 ng/mL). The turbidity was measured over time at 25°C and 350 nm, and the data were fitted to a one-phase association function. The fitted parameters for the pseudo-first order rate constant (k _*obs*_ ) ± 1 standard deviation are shown. Data represent two to three independent experiments. ^‡^ For WT, rates were very low precluding an accurate assessment of the pseudo-first order rate constant.

Subsequently, the conversion of the natural substrate fibrinogen by prothrombinase-activated prothrombin variants was assessed. Consistent with our observations on reduced clotting activity and impaired thrombin formation, a delayed fibrinogen conversion was observed for all activated prothrombin variants compared with wild type. This was indicated by a 7-fold reduced rate of fibrinogen conversion ( Fig. 8 ) and an approximately 10-fold prolongation in time to attain maximum turbidity ( Supplemental Fig. S5 (available in the online version only)). As such, modifications at the S4 subsite of (pro)thrombin negatively impact fibrinogen conversion. The rate of fibrinogen conversion by activated wild-type prothrombin was substantially reduced in the presence of increasing concentrations of dabigatran, with no detectable fibrinogen conversion at 1 μM dabigatran. In contrast, the rates of fibrin formation by activated prothrombin variants I174A, I174F, and KL3 were higher than those of wild type at all dabigatran concentrations tested. These findings make clear that S4 subsite modifications impair inhibition by dabigatran, leading to detectable fibrin formation at therapeutic dabigatran concentrations.

## Discussion

Through strategic modifications in or directly adjacent to the S4 subpocket of the thrombin active site, we have reduced thrombin's sensitivity to active site–targeted thrombin inhibitors. The extent of inhibitor resistance varied depending on the assay system. In a purified component assay, sensitivity to dabigatran decreased up to 24-fold, while a plasma-based assay showed a 2-fold reduction. MD simulations of unbound (apo) modified thrombin variants revealed these to comprise a larger inhibitor binding pocket relative to wild-type thrombin. In addition, computational analyses indicated reduced contributions of key interactive residues to inhibitor binding. Consequently, modified (pro)thrombin variants supported detectable fibrin formation at therapeutic dabigatran concentrations, demonstrating proof-of-concept for engineering thrombin variants that are resistant to direct thrombin inhibitors via S4 subsite modulation.

The S4 subsite, also known as the aryl binding site or distal (D) pocket, consists of Leu99 _c_ , Ile174 _c_ , and Trp215 _c_ and contributes directly to substrate binding. The direct thrombin inhibitors argatroban and dabigatran reversibly bind the active site by engaging the S4 subpocket in addition to ionic interactions with S1 subsite residue Asp189 _c_ . 38 39 Specifically, both argatroban and dabigatran comprise an aromatic structure that is positioned between Leu99 _c_ and Ile174 _c_ in the hydrophobic S4 subsite ( Fig. 1C ). 38 39 Modifications to the S4 subsite, including Ile174 _c_ substitutions and 99 _c_ -loop insertions, modulated inhibitor interactions, reducing sensitivity to argatroban and dabigatran by up to 4-fold and 24-fold, respectively. The IC _50_ values for dabigatran inhibition of wild-type thrombin assessed by amidolytic substrate conversion and peak thrombin formation were similar to earlier reported values (observed vs. reported *K*_i_ values: 4 vs. 5 nM for substrate conversion; and IC _50_ values: 532 vs. 570 nM for peak thrombin formation, respectively). 40 Argatroban has been reported to have an approximate 8-fold lower inhibitor constant for thrombin inhibition relative to dabigatran, 41 which is in line with our observations on peak thrombin formation. The 13-fold lower *K*_i_ value observed for argatroban inhibition of wild-type thrombin assessed by substrate conversion (observed vs. reported IC _50_ values: 3 vs. 40 nM 41 ) likely results from small differences in experimental conditions. Consistent with our observations, plasma-based thrombin generation assays required higher inhibitor concentrations than purified component assays to attain half maximal inhibition, due to plasma protein binding of thrombin inhibitors and feedback amplification of thrombin formation.

A molecular mechanism explaining the loss of inhibitor sensitivity following modification of the S4 subsite comes from MD simulations performed on both non-bound (apo) thrombin variants and inhibitor-bound thrombin variants. These computational analyses indicated that substituting Ile174 _c_ enlarged the inhibitor binding region and reduced interactions of residues located at or near the S4 subsite (173 _c_ and 174 _c_ ) with dabigatran and of key interactive residue Asp189 _c_ with argatroban. The 99 _c_ -loop insertion derived from human kallikrein-related peptidase KL3 was also projected to enlarge the inhibitor binding pocket, while weakening inhibitor interactions of residues 173 _c_ , 215 _c_ and 216 _c_ . Previously, active site residues Thr172 _c_ and Gly219 _c_ were observed to contribute to the correct conformation of dabigatran in the thrombin inhibitor binding pocket. 42 Our findings confirmed this observation, as interactions of residues 172 _c_ and 219 _c_ with dabigatran were observed for all thrombin variants, although these were projected to play a minimal to moderate role in inhibitor coordination, respectively. In summary, modifications at the S4 subsite likely disrupt the conformation of the active site, reducing critical intermolecular interactions and leading to impaired inhibitor binding. Computational assessments further revealed distinct key residues in dabigatran and argatroban coordination, explaining the thrombin variants' modestly impaired inhibition by argatroban compared with dabigatran.

Beyond inhibitor resistance, S4 subsite modifications also impaired protein C (approximately 2-fold reduction) and fibrinogen conversion (approximately 7-fold reduction). Fibrinogen interaction is mediated via thrombin exosite I, also known as anion binding site I comprising Lys36 _c_ , Lys67 _c_ , Lys73 _c_ , Arg75 _c_ , Arg78 _c_ , Lys109 _c_ , and Lys110 _c_ , which interacts with fibrinopeptides A and B. 43 S4 subsite residue Trp215 _c_ also directly interacts with fibrinogen. While thrombin's exosite I is also essential for recognizing and binding protein C, no role for the S4 subsite has been reported. 44 45 The Trp215 _c_ Ala substitution was shown to cause a 500-fold reduction in activity toward fibrinogen, 46 due to allosteric modulation of surface-exposed loops, the catalytic triad, and S1 pocket. 47 Whether similar mechanisms underlie the reduced procoagulant effects observed for Ile174 _c_ substitutions or 99 _c_ -loop insertions in the current study remains unclear. The observed impairment in Z-Gly-Gly-Arg-AMC conversion, also known as Cbz-Gly-Gly-Arg-AMC, suggests some level of perturbation of the S1–S4 subsites of the thrombin active site. In contrast, no defects were observed in the catalytic efficiency of H-D-Phe-Pip-Arg-pNA conversion. Whether this may be due to a more stringent coordination to the S1–S3 subsites or high conversion efficacy relative to Z-Gly-Gly-Arg-AMC would require further study. 48

**Fig. 9:**
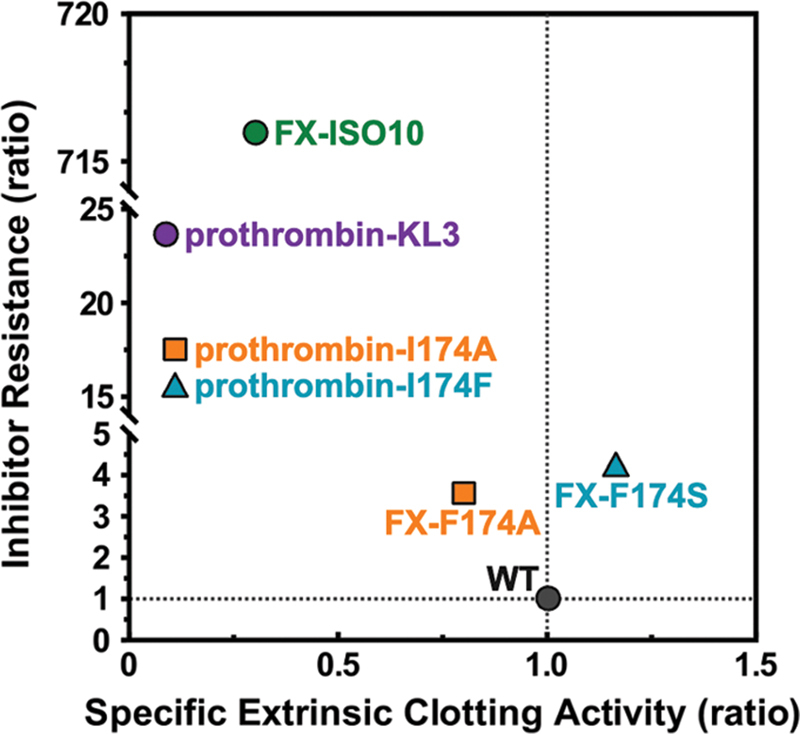
Inhibitor resistance versus clotting activity of S4 subsite modified prothrombin and FX variants. Inhibitor resistance is represented as the ratio of IC _50_ values of dabigatran inhibition of prothrombinase-activated prothrombin variants over wild-type prothrombinase-activated prothrombin obtained in a purified component system, or the ratio of IC _50_ values of apixaban inhibition of free FXa variants over wild-type FXa obtained in a purified component system. 22 23 The specific extrinsic clotting activity is represented as the ratio of the PT-based specific clotting activity of prothrombin variant over wild-type prothrombin or of FX variant over wild-type FX. 22 23 WT, wild-type; FX-ISO10 is referred to as FX-C in Verhoef et al. 22

Interestingly, the introduction of 99 _c_ -loop insertions KL3, KL10, and ISO10 produced various results. The KL3 insertion conferred partial inhibitor resistance with reduced clotting activity, while KL10 or ISO10 insertions substantially impaired both the extrinsic and intrinsic clotting activity. We speculate that this is the result of a perturbed activity toward fibrinogen as detailed above. In contrast, in FX(a) the ISO10 insertion was remarkably successful in terms of inhibitor resistance 22 ( Fig. 9 ). It should be noted that the 99 _c_ -loop sequence of ISO10 originates from an isoform of FX found in the venom and liver glands of the elapid snake *Pseudonaja textilis* , 24 49 50 which may contribute to the diminished impact of this loop when transplanted into prothrombin. Conversely, the 174 substitutions rendered (pro)thrombin more inhibitor resistant relative to similar modifications in FX(a) ( Fig. 9 ). Differences in response of homologous serine proteases to S4 modifications arise from structural divergence, 51 substrate specificity, 52 and allosteric effects. Thrombin's broader substrate interactions, mediated by exosites I and II, 51 further complicate these modifications.

Our study demonstrates that the conceptual approach of modifying prothrombin's S4 active site subpocket holds promise for potential advancement of novel direct thrombin inhibitor–bypassing agents in the future. However, several challenges must be addressed before clinical application. Given thrombin's role as a promiscuous serine protease, modifications may impact essential physiological processes. 53 54 Additionally, with circulating prothrombin levels at 1.4 µM, 55 significant concentrations of a bypassing agent would be required to demonstrate efficacy in vivo. Finally, non-inferiority to idarucizumab, the current reversal agent, must be demonstrated. Our findings highlight the non-interchangeable nature of modifying homologous serine proteases and underscore the need for computational approaches to predict DOAC-resistant thrombin variants while preserving catalytic activity.

## Notes

**What is known about this topic?**

- Modifications at or near the S4 subsite of the serine protease FXa previously reduced inhibition by direct FXa inhibitors.
- Expand the repertoire of specific strategies to reverse the anticoagulant effect of direct thrombin inhibitors.

**What does this paper add?**

- Modified S4 subsite prothrombin variants are resistant to direct thrombin inhibitors but display reduced procoagulant activity.
- Specific S4 subsite modifications do not exert the same effect between homologous serine proteases.
- Modifying the S4 active site subpocket holds promise for potential advancement of novel direct oral anticoagulant reversal agents.
