# Supplementary material for: Modifications of the Prothrombin Active Site S4 Subpocket Confer Resistance to Dabigatran
Source: Thromb Haemost. 2025 Mar 24;126(1):23–36. doi: 10.1055/a-2537-6037 (PMC12758957; doi:10.1055/a-2537-6037)
Supplement: Supplementary file 1 — Supplementary Material [file 10-1055-a-2537-6037-s24040170.pdf]

Supplementary Material S1

Supplemental Methods S1

Prothrombin Purification via Anion-exchange and Hydrophobic Affinity Chromatography

Conditioned media (15 L) was thawed at 37°C, filtered over a 0.45 µm polyethersulfone membrane, applied to a size 6 A ultrafiltration hollow fiber cartridge using an Äkta flux 6 instrument (Cytiva), diafiltrated to approximately 500 mL in 20 mM Hepes, 0.15 M NaCl, pH 7.4, and stored at −20°C. Following thawing at 37°C, the concentrate was applied at ambient temperatures to a 4.8 × 4 cm Q-Sepharose Fast Flow column (Cytiva) equilibrated in 20 mM Tris, 0.15 M NaCl, pH 7.4. After washing with the same buffer, bound protein was eluted with a linear 0.15 to 0.75 M NaCl gradient. Fractions containing prothrombin activity were stored at −80°C. Subsequent to thawing at 37°C, the fractions were pooled and dialyzed at 4°C, first for 3 hours to 1 mM EDTA, 20 mM Tris, pH 7.0 (5 L), next for 3 hours to 40 mM Na_2HPO_4/NaH_2PO_4, pH 6.8 (5 L), followed by overnight dialysis to the same buffer. The dialysate was centrifuged at 10,000 × g for 20 minutes at 4°C, and the supernatant was applied at ambient temperatures to a Bio-Scale CHT20-I hydroxyapatite column (Bio-Rad) equilibrated in 40 mM Na_2HPO_4/NaH_2PO_4, pH 6.8. Following washing with the same buffer, bound protein was eluted with a linear 40 to 400 mM Na_2HPO_4/NaH_2PO_4 gradient at a flow rate of 3 mL/min. Fractions containing prothrombin activity were analyzed employing SDS-PAGE analysis using the MES buffer system, stored at −80°C, pooled upon thawing at 37°C, precipitated by addition of 0.516 gr/mL ammonium sulfate (Sigma Aldrich, St Louis, Mo, USA) by stirring and overnight incubation at 4°C, collected by centrifugation (10,000 × g for 30 minutes at 4°C), dissolved in HBS supplemented with 0.1% (w/v) PEG8000 (dilution buffer) and 50% (v/v) glycerol, and stored at −20°C. The typical yield of fully γ-carboxylated recombinant prothrombin was 1 mg/L conditioned medium.

PDB Preparation for MD Simulations

To achieve the final thrombin–dabigatran topology, initially the serine protease heavy chain and the inhibitor in the thrombin–dabigatran analogue three-dimensional structure was retrieved from PDB ID 1KTS.[1] The Na^+ ion located in the sodium binding loop of thrombin was retrieved from PDB ID 4HFP[2] and positioned accordingly in the thrombin–dabigatran complex. The autolysis loop on thrombin was built in using Chimera UCSF 1.13.1[3] using the wild-type thrombin structure generated from AlphaFold as template. These AlphaFold generated structures were generated using the thrombin peptidase domain sequence retrieved from UniProt ID P00734 with default settings of AlphaFold. The complex achieved in Chimera was minimized in implicit solvation and using maximally 5,000 cycles using sander. At the 100th cycle, minimization was switched from steepest descent to conjugate gradient. The additional ethyl moiety found in the analogue of dabigatran was truncated to achieve the structure of dabigatran bound to thrombin. Hydrogen atoms were added to dabigatran using reduce command provided in AmberTools23. The protonation state of dabigatran was selected to represent the anion form of the ligand.[4] Point mutations were implemented by running the LEaP program after manually removing the side chain and renaming the backbone of the I174 residue to ALA or PHE in the final complex structure. The KL3-loop of the thrombin-KL3 variant was modeled using Chimera and the PDB structure generated from AlphaFold for the structure. In similar fashion, the topologies regarding thrombin–argatroban or variants-argatroban complex were generated starting from the PDB ID 1DWC.[5] The parameter and topology files used during the MD simulations were prepared as shown in the LEaP file (►**Supplemental Table S1**).

Supplemental Table S1 Input files for LEaP

LEaP file
source leaprc.protein.ff19SB<br>source leaprc.water.tip3p<br>source leaprc.gaff<br>LIG = loadmol2 LIG_clean_H.mol2<br>loadamberparams LIG.frcmod<br>ramp = loadpdb PDB_amber.pdb<br>addIons ramp Cl⁻ 0<br>solvateOct ramp TIP3PBOX 14.0<br>addIonsRand ramp Na⁺ 25 Cl⁻ 25<br>saveamberparm ramp inp.prmtop inp.inpcrd<br>check ramp<br>savepdb ramp PDB_postLEap.pdb<br>quit

Amber forcefield 19SB[6] was used to describe thrombin and the variants, and the General Amber Forcefiel[7] was used to describe dabigatran or argatroban. Dabigatran or argatroban parameter modifications and mol2 files were generated using standard Amber protocols. The protein–ligand complex was solvated in an Octahedron box using a TIP3P water model.[8] For argatroban simulations, thrombin variants comprising the I174I, I174A, or I174F substitutions were solvated with 11373, 11423, or 11256 water molecules, respectively. The KL3 variant was solvated in 14192 molecules. For dabigatran simulations, thrombin variants comprising the I174I, I174A, or I174F substitutions were solvated with 10400, 10424, or 10604 water molecules, respectively. The KL3 variant was solvated in 12379 water molecules. For apo simulations, thrombin variants comprising the I174I, I174A, or I174F substitutions were solvated with 9648, 12521, or 9622 water molecules, respectively. The KL3 variant was solvated in 12719 water molecules. Sodium and chlorine ions were added to a 0.15 M NaCl concentration.

 © 2025. The Author(s).

Supplemental Table S2 Input files for energy minimization and MD simulations

First energy minimization	Second energy minimization	Heating	NPT equilibration	NVT equilibration	MD simulation (production)
&cntrl<br>imin = 1,<br>ntb = 1,<br>cut = 12.0,<br>irest = 0,<br>ntmin = 1,<br>ncyc = 100,<br>maxcyc = 1000,<br>igb = 0, saltcon = 0.15,<br>ntpr = 50,<br>ntwx = 50,<br>/	&cntrl<br>imin = 1,<br>ntb = 1,<br>cut = 12,<br>irest = 0,<br>ntmin = 1,<br>ncyc = 250,<br>maxcyc = 5000,<br>igb = 0,<br>saltcon = 0.15,<br>ntpr = 500,<br>ntwx = 500,<br>ntwf = 500,<br>ntxo = 2,<br>/	&cntrl<br>ntt = 3,<br>gamma_ln = 5.0,<br>ntc = 2,<br>ntf = 2,<br>ntb = 1,<br>cut = 8.0,<br>dt = 0.002,<br>nstlim = 1500000,<br>ig = −1,<br>ntwr = 10000,<br>ntwx = 10000,<br>ntpr = 10000,<br>ioutfm = 1,<br>iwrap = 1,<br>/	&cntrl<br>ntt = 3,<br>gamma_ln = 2.0,<br>ntc = 2,<br>ntf = 2,<br>ntb = 2,<br>ntp = 1,<br>barostat = 2,<br>pres0 = 1.0,<br>taup = 1.0,<br>cut = 8.0,<br>dt = 0.002,<br>nstlim = 4000000,<br>ig = −1,<br>ntwr = 10000,<br>ntwx = 10000,<br>ntpr = 10000,<br>ioutfm = 1,<br>iwrap = 1,<br>igb = 0,<br>saltcon = 0.15,<br>irest = 1,<br>ntx = 5,<br>/	&cntrl<br>ntt = 3,<br>gamma_ln = 2.0,<br>ntc = 2,<br>ntf = 2,<br>ntb = 1,<br>cut = 8.0,<br>dt = 0.002,<br>nstlim = 10000000,<br>ig = −1,<br>ntwr = 10000,<br>ntwx = 10000,<br>ntpr = 10000,<br>ioutfm = 1,<br>iwrap = 1,<br>igb = 0,<br>saltcon = 0.15,<br>irest = 1,<br>ntx = 5,<br>/	&cntrl<br>ntt = 3,<br>gamma_ln = 2.0,<br>ntc = 2,<br>ntf = 2,<br>ntb = 1,<br>cut = 8.0,<br>dt = 0.002,<br>nstlim = 50000000,<br>ig = −1,<br>ntwr = 5000,<br>ntwx = 5000,<br>ntpr = 5000,<br>ioutfm = 1,<br>iwrap = 1,<br>igb = 0,<br>saltcon = 0.15,<br>irest = 1,<br>ntx = 5,<br>/

A modified version of the protocol proposed by Cruzeiro et al was implemented.[8] During the preparatory steps for MD simulation the cut-off for non-bonded interactions was set to 12 Å during minimization and decreased to 8 Å for the other steps. The first energy minimization was performed with maximally 1,000 cycles using sander at the 100th cycle; minimization was switched from steepest descent to conjugate gradient. The second energy minimization was performed in a similar manner, but the maximum number of cycles was set to 5,000; the process was run using pmemd.cuda and the switch to conjugate gradient was performed at the 250th cycle. After energy minimization, the system was gradually heated from 10 to 300K over a span of 0.6 nanoseconds, succeeded by a 2.4 nanoseconds simulation at 300K to achieve thermal equilibration. The NPT equilibration was performed in an 8 nanoseconds simulation. Following this, NVT equilibration was conducted over an 8 nanoseconds period. Production MD simulations extended over 100 nanoseconds with coordinate frames written out every 0.2 nanoseconds.

 © 2025. The Author(s).

Supplemental Table S3 Specific extrinsic or intrinsic clotting activity of purified prothrombin variants

	Specific PT activity (U/mg)	Specific APTT activity (U/mg)
Prothrombin-WT	7.5 ± 0.09	8.7 ± 0.79
Prothrombin-I174A	0.8 ± 0.24[a]	1.5 ± 0.19[a]
Prothrombin-I174F	0.8 ± 0.25[a]	1.7 ± 0.27[a]
Prothrombin-KL3	0.7 ± 0.24[a]	1.4 ± 0.50[a]
Prothrombin-KL10	0.6 ± 0.19[a]	0.6 ± 0.18[a]
Prothrombin-ISO10	0.4 ± 0.16[a]	0.4 ± 0.21[a]

Abbreviation: WT, wild-type.
Notes: The specific extrinsic or intrinsic clotting activity was determined as described in "Methods" using NPP as reference comprising 1 U/mL prothrombin activity and expressed in units per mg (U/mg) purified protein. The data are provided as mean values ± 1 standard deviation of at least two independent experiments.
[a]$p < 0.0001$ according to one-way ANOVA analysis in comparison with wild-type prothrombin.

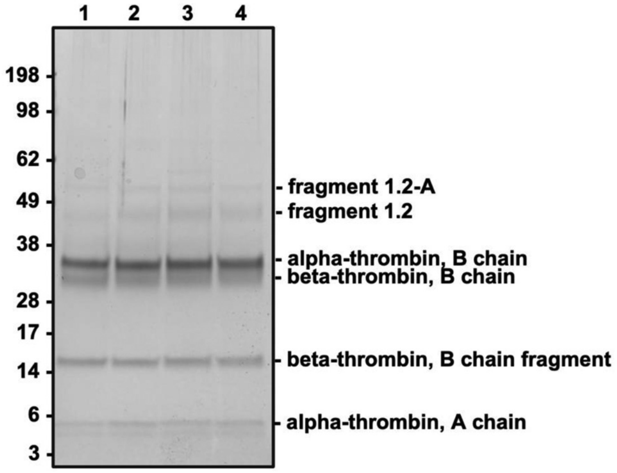

Supplemental Fig. S2 Prothrombinase activation of prothrombin variants. Purified prothrombin variants activated with preassembled prothrombinase as detailed in "Methods" and subjected to SDS-PAGE analysis (1 µg/lane) under non-reducing conditions and visualized by Coomassie staining. Lane 1, wild-type prothrombin; lane 2, prothrombin-KL3; lane 3, prothrombin-I174A; lane 4, prothrombin-I174F. The protein bands and apparent molecular weights (kDa) of the standards are indicated. All activated variants migrate predominantly as α-thrombin. The proportion of β-thrombin, which results from limited autoproteolysis, is similar for all activated variants. While the kinetic parameters for synthetic substrates are similar to those of α-thrombin, β-thrombin has decreased affinity for thrombomodulin, reduced reactivity toward protein C, and diminished fibrinogen clotting activity compared with α-thrombin.[9,10]

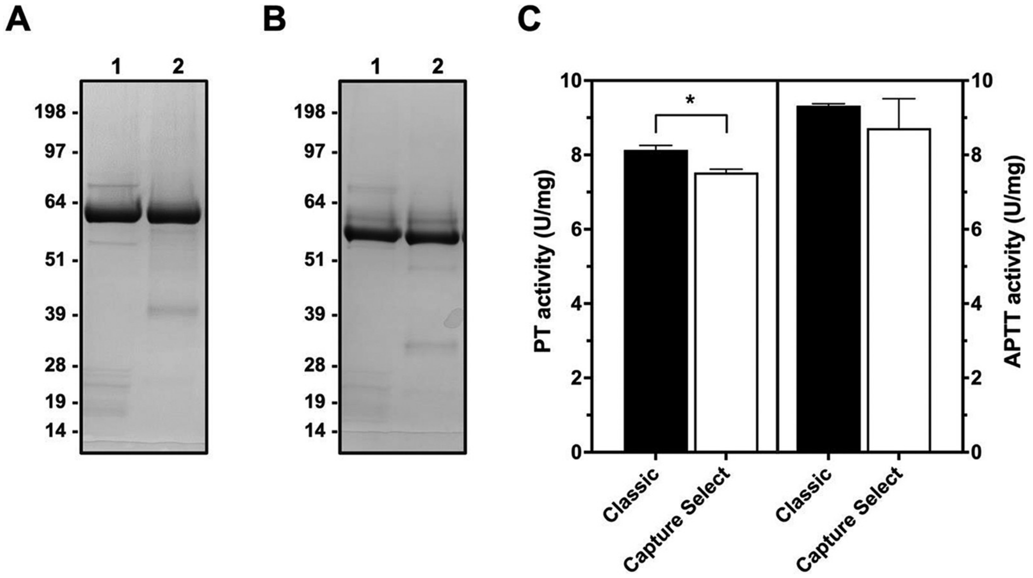

Supplemental Fig. S1 Prothrombin preparations purified employing anion-exchange and hydrophobic affinity chromatography versus the CaptureSelect Prothrombin affinity chromatography. (**A, B**) SDS-PAGE analysis of wild-type prothrombin (3 µg/lane) purified using either anion-exchange and hydrophobic affinity chromatography purification steps ("Classic," lanes 1) as detailed in "Supplemental Methods" or the CaptureSelect Prothrombin affinity chromatography procedure ("Capture Select," lanes 2) under reducing (**A**) or non-reducing (**B**) conditions employing the MOPS buffer system and visualized by Coomassie staining. The apparent molecular weights (kDa) of the standard are indicated. (**B**) The specific clotting activity initiated by the extrinsic or intrinsic pathway employing a prothrombin-specific prothrombin time (PT)-based assay (columns on the left) or prothrombin-specific activated partial thromboplastin time (APTT)-based assay (columns on the right), respectively, was determined for the wild-type prothrombin protein preparations purified using the Classic or CaptureSelect method. $^{*}p < 0.05$ according to two-sample *t*-test.

 © 2025. The Author(s).

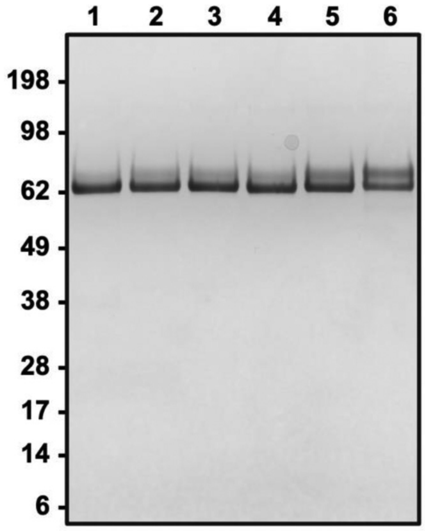

Supplemental Fig. S3 Non-reduced SDS-PAGE analysis of prothrombin variants. SDS-PAGE analysis of purified prothrombin variants (3 μg/lane) under non-reducing conditions and visualized by Coomassie staining. Lane 1, wild-type prothrombin; lane 2, prothrombin-I174A; lane 3, prothrombin-I174F; lane 4, prothrombin-KL3; lane 5, prothrombin-KL10; lane 6, prothrombin-ISO10. The apparent molecular weights (kDa) of the standards are indicated.

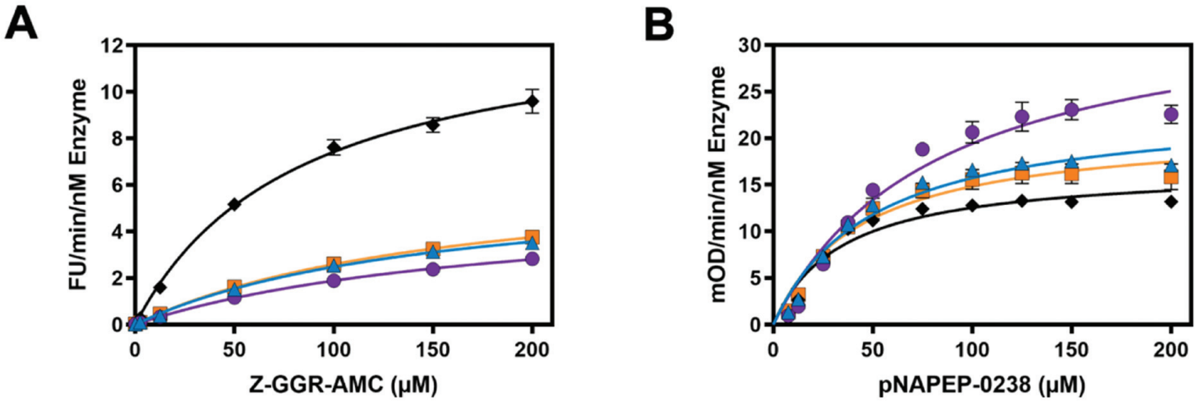

Supplemental Fig. S4 Peptidyl substrate conversion by activated prothrombin variants in a purified system. Prothrombinase-activated variants (5 nM) were incubated with increasing concentrations (0–200 μM) of fluorescent substrate Z-GGR-AMC (**A**) or chromogenic substrate pNAPEP-0238 (**B**). Peptidyl substrate conversion was assessed as described in "Methods"; fitted values ± 1 standard deviation of the induced fit are shown. The data of two to three independent experiments are shown. FU, fluorescent unit; OD, optical density at 405 nm.

 © 2025. The Author(s).

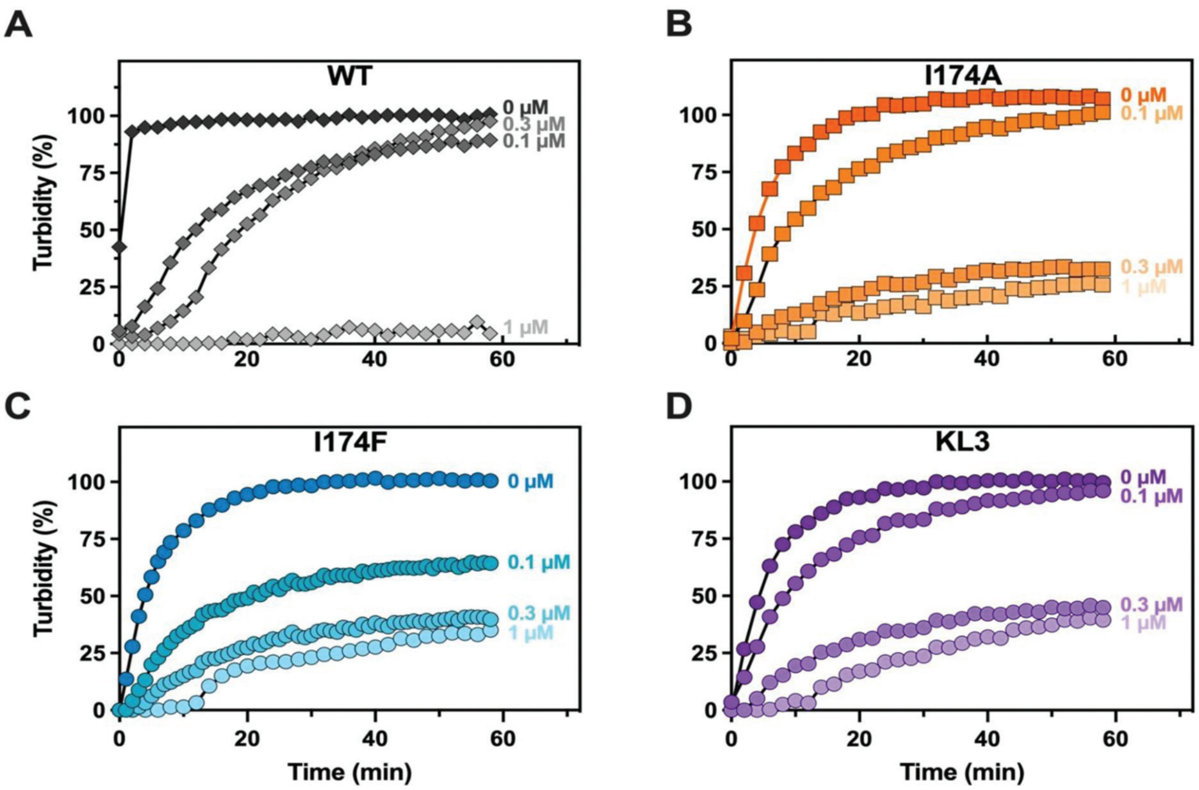

Supplemental Fig. S5 Fibrinogen conversion by prothrombinase-activated prothrombin variants. Prothrombinase-activated variants (0.045 μM) prothrombin-WT (black diamonds) (**A**), prothrombin-I174A (orange squares) (**B**), prothrombin-I174F (teal triangles) (**C**), or prothrombin-KL3 (purple circles) (**D**) were incubated with fibrinogen (0.9 μM) and dabigatran (0–1 μM). The turbidity was measured over time at 25°C and 350 nm. Data represent two to three independent experiments.

Supplemental References

1 Hauel NH, Nar H, Priepke H, Ries U, Stassen J-M, Wienen W. Structure-based design of novel potent nonpeptide thrombin inhibitors. J Med Chem 2002;45(09):1757–1766

2 Pozzi N, Chen Z, Zapata F, et al. Autoactivation of thrombin precursors. J Biol Chem 2013;288(16):11601–11610

3 Pettersen EF, Goddard TD, Huang CC, et al. UCSF Chimera—a visualization system for exploratory research and analysis. J Comput Chem 2004;25(13):1605–1612

4 Case DA, Duke RE, Walker RC, et al. Amber 2022. San Francisco: University of California; 2022

5 Banner DW, Hadváry P. Crystallographic analysis at 3.0-A resolution of the binding to human thrombin of four active site-directed inhibitors. J Biol Chem 1991;266(30):20085–20093

6 Tian C, Kasavajhala K, Belfon KAA, et al. ff19SB: amino-acid-specific protein backbone parameters trained against quantum mechanics energy surfaces in solution. J Chem Theory Comput 2020;16(01):528–552

7 Wang J, Wolf RM, Caldwell JW, Kollman PA, Case DA. Development and testing of a general amber force field. J Comput Chem 2004;25(09):1157–1174

8 Cruzeiro VWD, Amaral MS, Roitberg AE. Redox potential replica exchange molecular dynamics at constant pH in AMBER: implementation and validation. J Chem Phys 2018;149(07):072338

9 Bezeaud A, Guillin MC. Enzymic and nonenzymic properties of human beta-thrombin. J Biol Chem 1988;263(08):3576–3581

10 Soslau G, Goldenberg SJ, Class R, Jameson B. Differential activation and inhibition of human platelet thrombin receptors by structurally distinct alpha-, beta- and gamma-thrombin. Platelets 2004;15(03):155–166

 © 2025. The Author(s).
